# Physiological confounders of renal blood flow measurement

**DOI:** 10.1007/s10334-023-01126-7

**Published:** 2023-11-16

**Authors:** Bashair Alhummiany, Kanishka Sharma, David L. Buckley, Kywe Kywe Soe, Steven P. Sourbron

**Affiliations:** 1 Department of Biomedical Imaging Sciences, University of Leeds, Leeds, LS2 9NL UK; 2 Department of Imaging, Infection, Immunity and Cardiovascular Disease, The University of Sheffield, Sheffield, UK

**Keywords:** Renal blood flow, Kidney, Perfusion, Within-subject variation, Between-subject variation

## Abstract

**Objectives:**

Renal blood flow (RBF) is controlled by a number of physiological factors that can contribute to the variability of its measurement. The purpose of this review is to assess the changes in RBF in response to a wide range of physiological confounders and derive practical recommendations on patient preparation and interpretation of RBF measurements with MRI.

**Methods:**

A comprehensive search was conducted to include articles reporting on physiological variations of renal perfusion, blood and/or plasma flow in healthy humans.

**Results:**

A total of 24 potential confounders were identified from the literature search and categorized into non-modifiable and modifiable factors. The non-modifiable factors include variables related to the demographics of a population (e.g. age, sex, and race) which cannot be manipulated but should be considered when interpreting RBF values between subjects. The modifiable factors include different activities (e.g. food/fluid intake, exercise training and medication use) that can be standardized in the study design. For each of the modifiable factors, evidence-based recommendations are provided to control for them in an RBF-measurement.

**Conclusion:**

Future studies aiming to measure RBF are encouraged to follow a rigorous study design, that takes into account these recommendations for controlling the factors that can influence RBF results.

## Introduction

Renal blood flow (RBF) has an important role in providing high capillary pressure to drive glomerular filtration and maintain oxygen supply to meet the demands of renal metabolism. A well-recognized feature of the renal circulation is its ability to maintain RBF at a relatively constant level as a result of autoregulation [1]. This mechanism is essential for preserving body fluid balance and protecting the glomerular capillaries from an increase in blood pressure. Renal autoregulation does not imply that RBF is unchangeable, as RBF is known to change remarkably with daily activities [2, 3]. This is because the kidney actively participates in blood pressure regulation [4] through baroreceptor reflex and the release of vasoactive agents [5] which ultimately modulate renal haemodynamics.

Alterations in renal microcirculation play a critical role in the pathophysiological mechanisms of renal disease [6]. Measurement of RBF is therefore considered an important biomarker in the evaluation of renal function. However, a significant problem in the interpretation of RBF is the large heterogeneity of normal reported values due to combination of measurement error and inherent physiological changes of RBF [7]. While it is difficult to distinguish between the two effects, understanding and quantifying potential physiological factors can promote standardization and reduce variability in RBF measurements. Such standardization is essential to ensure that disease-related changes are not confounded by physiological events.

Recently, a consensus-based project has outlined a number of technical recommendations to improve the standardization of renal biomarkers such as RBF, perfusion and blood oxygen level dependent (BOLD) magnetic resonance imaging (MRI) [8–10]. The results highlighted a lack of consensus among experts concerning aspects of patient preparation due to incomplete understanding of the impact of physiological variability.

The aim of this review is to develop a practical reference for the preparation and interpretation of RBF values. For this purpose, a literature review was conducted to identify and assess a wide range of factors that can contribute to the variability in RBF in heathy humans. Based on brief discussion of the variability in the applied techniques and a comprehensive analysis of the physiological variability in the literature, evidence-based recommendations will be formulated for future studies aiming to achieve a more standardized approach for RBF quantification.

## Materials and methods

### Scope

Different biomarkers quantify the amount of blood delivered to the kidney. Renal blood flow (RBF, expressed in mL/min) measures the delivery of blood at the macrovascular level, whereas tissue perfusion (expressed in mL/min/100 mL) measures delivery to the capillaries at the microvascular level. Measurements of RBF are often indexed to body surface area (BSA) and presented per 1.73 m^2^, thereby providing comparable estimates between different populations.

Different methods are available to measure RBF [Clearance technique, Radionuclide scintigraphy, Doppler ultrasound (US), and Phase contrast MRI (PC)], or perfusion [Positron emission tomography (PET), Dynamic contrast enhanced (DCE) and Arterial spin labelling (ASL)]. Reference values tend to be dependent on the measurement methodology used and are summarized in Table 1. All these methods are considered in this review, assuming that relative changes under influence of confounders are comparable even if absolute values are not. Analysis of this review was based on RBF (mL/min) with renal perfusion studies only interpreted in the results section.

**Table 1:** Typical renal hemodynamic measurements obtained using available techniques in humans with normal kidney function

Technique	References	*N* (F/M)	Age	Renal blood flow (RBF)^a^		Remarks
				mL/min/1.73 m^2^	mL/min	
Clearance technique	Goldring et al*.* [178]	43 (0/43)	39 ± 12	1189 ± 242	1205 ± 245	Diodrast (continuous infusion)
	Bolomey et al*.* [179]	18 (5/13)	41 ± 10	1052 ± 236	1014 ± 228	PAH (continuous infusion)
Radionuclide scintigraphy	Esteves et al*.* [180]	106 (62/44)	40 ± 10.8	321 ± 69	356 ± 76	^99m^Tc-MAG_3_ (camera clearance)
Doppler ultrasound	Greene et al*.* [181]	16 (6/10)	28 ± 8	790 ± 125	800 ± 127	Average velocity profile
Phase contrast MRI	Bax et al*.* [19]	20	29 ± 5	–	1133 ± 268	Cine (2D) imaging
	Eckerbrom et al*.* [25]	28 (15/13)	24 ± 5.3	963 ± 160	1013 ± 169	2D imaging with cardiac gating

				Renal perfusion (mL/min/100 mL)		
				Cortex	Medulla	
Positron emission tomography	Nitzsch et al*.* [182]	20 (4/16)	29 ± 9	470 ± 28	–	^15^O-water
	Normand et al*.* [183]	10 (0/10)	22 ± 3.7	329 ± 65	–	^15^O-water
Dynamic contrast enhanced MRI	Wu et al*.* [184]	19 (7/12)	41 ± 15	272 ± 60	122 ± 30	Magnevist contrast agent
	Eikefjord et al*.* [185]	20 (16/4)	25 ± 4.5	345 ± 84	–	Dotarem contrast agent
Arterial spin labelling (ASL)	Wu et al*.* [184]	19 (7/12)	41 ± 15	227 ± 30	101 ± 21	Pseudo-continuous ASL
	Eckerbrom et al*.* [25]	28 (15/13)	24 ± 5.3	290 ± 63	91 ± 14	Pulsed ASL

^a^Conversion between RBF units was performed using body surface area provided in the paper

### Literature search and selection

An initial exploratory search was performed using Pubmed, Ovid MEDLINE and Web of Science databases with the terms physiological, biological, variability and ‘renal circulation’. Articles obtained from this search were used to determine a list of physiological factors that cause changes in RBF. The identified factors focussed both on normal physiological changes that cause within- or between-subject variation in RBF. Each identified factor was searched individually to include studies up to June 2021. The search terms used for each influencing factor are provided in Table 2. Reference lists from acquired articles were also searched for inclusion of relevant studies. English, full-text articles reporting perfusion, blood and/or plasma flow in adult human subjects were included in this review.

**Table 2:** Search terms for the potential factors influencing renal blood flow

RBF terms		Influencing factor	Search terms	Number of articles
‘Renal blood flow’	AND	Age	Age	1190
‘Renal plasma flow’			Lifespan	
‘Renal circulation’			Elderly	
‘Renal perfusion’		Gender	Gender	142
			Sex	
			Dimorphism	
		Race	Race	57
			Ethnicity	
		BMI	‘Body mass index’	151
			Lean	
			Overweight	
			Obese	
		Circadian rhythm	‘Circadian rhythm’	27
			‘Circadian cycle’	
			‘Biological clock’	
		Pregnancy	Pregnan*	279
			Gestation	
			Matern*	
		Menstrual cycle	‘Menstrual cycle’	18
			‘Follicular phase’	
			‘Luteal phase’	
		Food intake	Food	203
			Meal	
			Ingest*	
			Eat*	
		Fluid intake	Water	1142
			Hydrat*	
			Fluid	
		Smoking	Nicotine	35
			Smok*	
			Cigarette*	
		Caffeine	Caffeine	62
			Coffee	
			‘Energy drink*’	
			‘Soft drink*’	
		Alcohol	Alcohol	233
			Drink*	
			Beer	
		Altitude	Altitude	20
			Climb*	
		Exercise	Exercise	273
			‘Physical activity’	
			Training	
		Mental stress	‘Mental stress’	94
			Anxiety	
			Depression	
		Thermal stress	‘Heat stress’	80
			*Thermia	
		Medication	Medication*	2985
			Drug*	
			Treatment*	
Combined with OR			Combined with OR	

^*^Truncation command to search for the root of the free-text word with any alternative ending

### Data synthesis

The magnitude of change was determined for each factor as the absolute (mL/min) and relative (%) difference of baseline RBF values. Results are presented as mean (and range) of absolute and relative change in RBF across studies. Since renal dysfunction can modulate the renal response to some physiological factors, only studies reporting on subjects with normal kidney function were included in the analysis. When RPF was reported, a conversion to RBF was made assuming a haematocrit value of 42% known from the literature [11], except where haematocrit was reported in the original study.

## Results

The search terms yielded a total of 6987 articles, after removing duplicates. Following abstract and title screening and assessing for eligibility, 215 full-text articles were reviewed. A total of 162 original studies were summarized and included in this review; of which 110 studies reported quantitative measurement for the absolute and/or relative change. These studies investigated the effect of 24 potential factors on renal circulation.

**Fig. 1:**
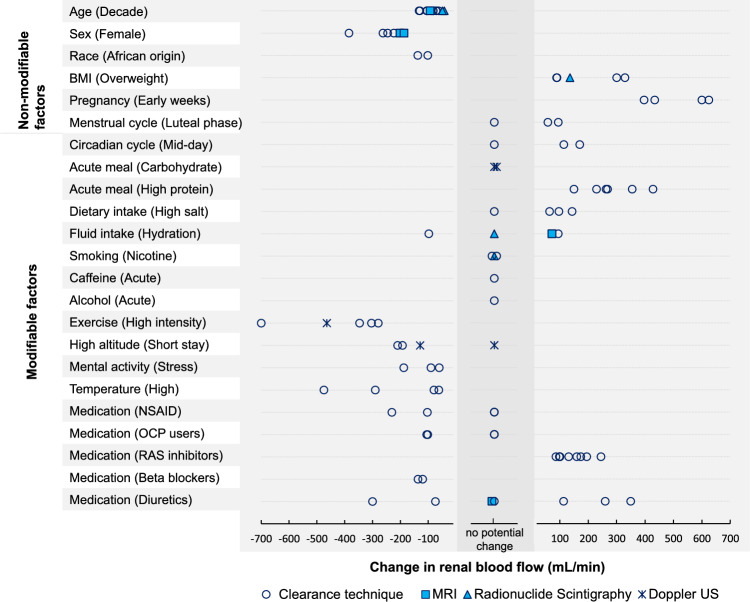
The absolute change in renal blood flow for all physiological confounders. Each point refers to measurement reported by one empirical study and coded according to the applied method

**Fig. 2:**
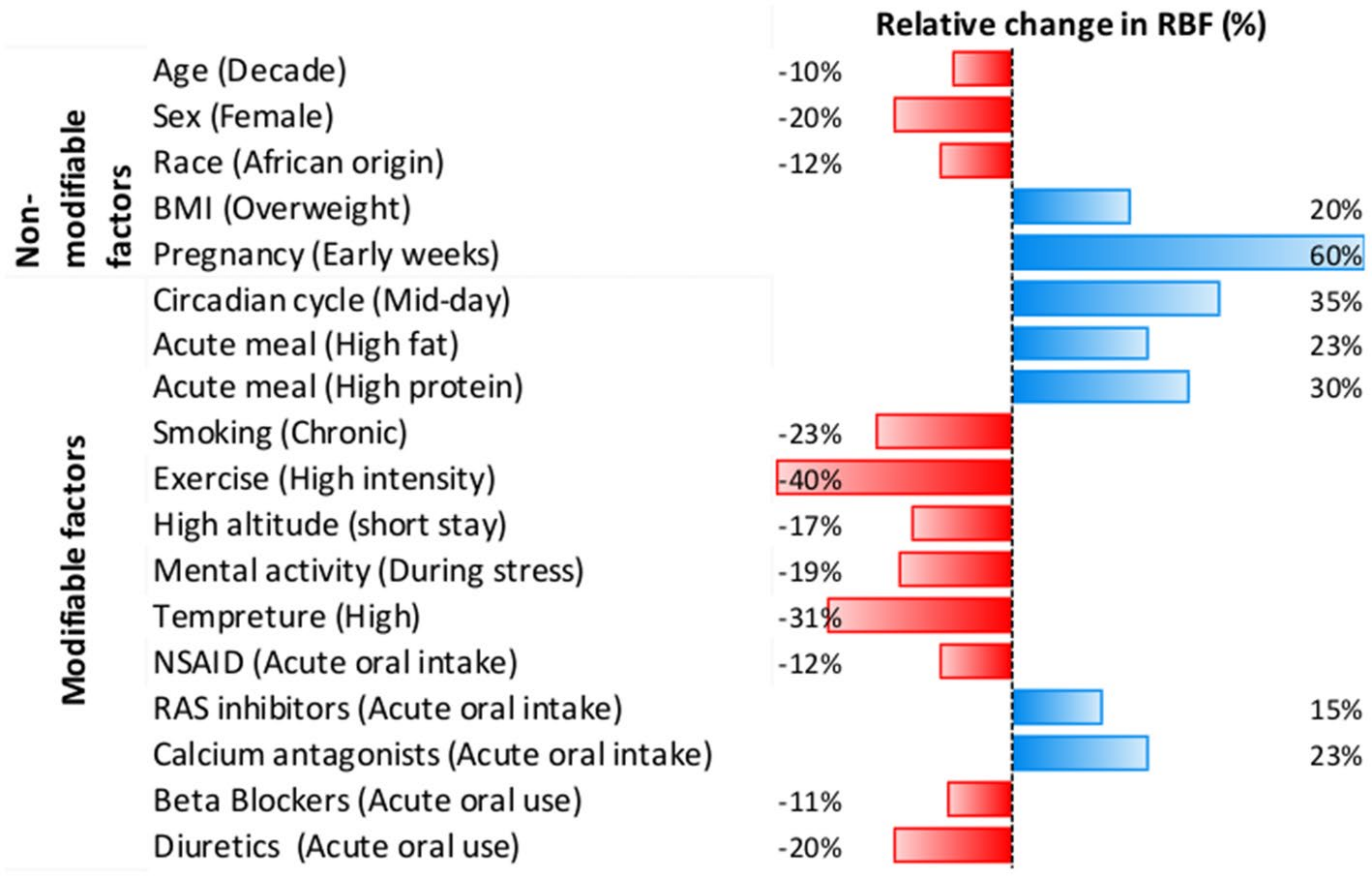
The relative change in renal blood flow (%) averaged across studies. Only factors with consistent impact reported in the literature are presented

The factors were divided into non-modifiable and modifiable. Non-modifiable factors essentially compromise the demographic characteristics of a population, such as age, sex, and race. While these factors cannot be manipulated in an experiment, acknowledging their impact on the measured RBF means that researchers can account for those in the study design or data analysis. The modifiable factors include a range of activities which can be controlled for in an experiment. Figure 1 shows the absolute change in RBF obtained from each study for all identified factors. The estimated absolute and relative change in RBF average across studies were summarized in Table 3 and presented in Fig. 2.

**Table 3:** Summary of all physiological confounders of renal blood flow and possible ways to control

Influencing factor	RBF response	Change in RBF % (and absolute)	How to control
*Before/during measurement*			
Acute meal (High fat) [59]	↑	+ 23%	Provide standard meal/use questionnaire to record last meal
Acute meal (Carbohydrate) [60, 61]	=	=	
Acute meal (High protein) [62–68]	↑	+ 30% (260 mL/min)	
Fluid intake (Hydration) [83]	↕		Normal hydration protocol
Nicotine (acute smokers) [89]	=	=	No restriction
Caffeine (Acute) [93, 94]	=	=	
Alcohol (Acute) [92]	=	=	
Exercise (High intensity) [96–100]	↓	− 40% (− 450 mL/min)	Avoid heavy physical exercise on the day
High altitude (Short stay) [115, 116]	↓	− 17% (− 177 mL/min)	Use questionnaire to record recent exposure to high altitude
Circadian cycle (Mid-day) [3, 54–56]	↑	+ 35% (283 mL/min)	Schedule repeat scans at fixed time of the day
Pregnancy (Early weeks) [44–47]	↑	+ 60% (514 mL/min)	Use questionnaire to record menstrual health and history
Menstrual cycle (Luteal phase) [48–51]	↑	+ 8% (77 mL/min)	
Mental activity (During stress) [121, 122]	↓	− 19% (− 135 mL/min)	Ensure patient feels comfortable. Record feelings of anxiety
Temperature (High) [97, 128]	↓	− 31% (− 383 mL/min)	Measure body temperature
NSAID (Acute oral intake) [137–139]	↓	− 12% (122 mL/min)	Record regular use of treatments. Withdraw acute use of medication when possible
OCP (Regular use) [153, 154]	↓	− 10% (102 mL/min)	
RAS inhibitors (Acute oral intake) [148, 158, 160, 162, 163, 186]	↑	+ 15% (282 mL/min)	
Calcium antagonists (Acute oral intake) [159]	↑	+ 23%	
Beta blockers (Acute oral intake) [158, 167]	↓	− 11% (− 128 mL/min)	
Diuretics (Acute oral intake) [169]	↓	− 20% (− 187 mL/min)	
*Interpreting the measurement*			
Age (> 40 years) [13, 16, 17, 19]	↓	− 10% (− 85 mL/min) per decade	Include in statistical analysis model
Sex (Female) [16, 19, 23]	↓	− 20% (− 243 mL/min)	
BMI (Overweight) [39, 40]	↑	+ 20% (105 mL/min)	
*Additional information*			
Race [30]	?		For between-subjects comparison. Use questionnaire to record individual habits
Diet habits (low salt) [75–77]	↓	− 10% (− 100 mL/min)	
Diet habits (low protein) [71, 72]	↓	− 10% (− 94 mL/min)	
Diet habits (Plant-based) [69]	↓	− 12% (− 132 mL/min)	
Smoking habits (chronic smokers) [90]	↓	− 23%	
Drinking habits	?		
Exercise habits (fit) [107]	?		
High altitude (natives) [118]	↓	− 28% (− 288 mL/min)	
Depression/anxiety	?		

↑ Increase RBF; ↓ Decrease RBF; ↕ Both increase and decrease have been reported; = No change in RBF; ? a potential factor (not sufficiently studied)

### Non-modifiable factors

#### Age

The kidney undergoes multiple changes with advanced age, including reduced renal function [12], decreased kidney size, and alterations in blood flow. Changes in RBF with normal ageing are well-documented and have been reported using different methods. Davies and Shock [13] were one of the first to report the decline in resting RBF in adults after the age of 40. In this study, baseline measurements using clearance methods were compared in male subjects aged between 20 and 90 years old. Differences in RBF were observed between age groups for each decade with the largest reduction (53%) between the youngest and the oldest groups from a mean of 1170 to 433 mL/min. This observation has consistently been reported by other investigators using PAH clearance [14–17], ^99m^Tc-MAG_3_ scintigraphy [18] and similarly using PC-MRI [19, 20]. Renal cortical perfusion estimated with ASL and DCE showed lower values in the older adults and negative association with advanced age [20–22]. Collectively, RBF was found to decrease after the age of 40 by − 10% each decade with an average decline of − 85 mL/min per decade (range from − 49 to − 106 mL/min) between different studies [13, 16, 19]. The effect of age is important and should therefore be considered when interpreting RBF between subjects of different age groups.

#### Sex

The total RBF in women was found to be − 20% (range − 16 to − 23%) lower than men by an average of − 243 mL/min (range − 187 to − 385 mL/min) [16, 19, 23–25]. The effect of gender on the renal haemodynamics appears to be driven by the protective action of oestrogen in women during reproductive age [26]. As a result, an interaction between age and gender was demonstrated, where gender-related differences were found to disappear after the age of 60 [16, 19]. The gender effect on RBF can also be secondary to the difference in body size and kidney volume between men and women [27]. The findings reported by Berg [23] suggest no differences when the measurement was indexed to 1.73 m^2^ BSA. While it remains unclear whether there is a truly physiological difference, the gender effect appears to be important and should be considered.

#### Race

Race has long been considered a critical variable in the estimation of glomerular filtration rate (eGFR). The misuse of race in the assessment of renal function is subject to an ongoing debate [28], and a replacement of current eGFR equations is under evaluation [29]. However, studies investigating racial differences for RBF are limited. Using PAH clearance, RPF of African Americans was found to be 16% lower than age-matched Caucasians when participants followed a high-salt diet [30]. The difference in RPF between the two groups disappeared when switching to a low-salt diet in a subsequent study [31]. These data suggest an underlying physiological difference between the two ethnic groups, which involve a blunted renal response to angiotensin-II receptor antagonist in the kidneys of African Americans [32]. There is also some evidence pointing towards the involvement of genetic factors, whereby specific gene variants appear to be associated with RPF variations in individuals of African descent [33]. Future studies on sufficiently large population and including other racial groups are necessary to better understand how race, social and genetic factors interact to affect kidney function.

#### Body mass index

The effect of body mass index (BMI) on renal haemodynamics is not specific to obesity or being overweight as it appears across wide BMI ranges. Comparing lean (BMI: 18–25 kg/m^2^) and overweight subjects (BMI: 25–35 kg/m^2^), with normal kidney function, showed a marked positive correlation with renal circulation [25, 34], and an increase in RBF on average by 20% (range 13–31%) with higher BMI [34–36]. The relation between BMI and RBF persists even when adjusted for age and sex [37, 38]. A reduction in RBF with weight loss following bariatric surgery has also been reported [39, 40]. However, renal perfusion measured using ^15^O-H_2_O PET in both cortex and medulla was not affected by changes in weight between normal and obese [40]. Of note, correcting RBF for body dimensions (expressed per 1.73 m^2^ BSA) was found to be negatively associated with BMI [37, 38, 41]. Indexing for height has been suggested as an alternative approach [42], in which the association with RBF was found to be eradicated and therefore, make the measurement more comparable between individuals. However, this alternative way of indexing was not widely adopted as it would lead to the same error as BSA in populations of variable height measurements. Overall, the effect of BMI is apparent and should be considered when comparing RBF between subjects with variable body dimensions.

#### Pregnancy and menses

Major changes in RBF were noted during pregnancy, which can occur as early as the sixth gestational week [43]. Serial studies using PAH clearance performed on pregnant women showed substantial increase in RBF during the first trimester by an average of 514 mL/min (range 434–625 mL/min) [44–47] reaching maximal level at ~ 12 gestational week [43]. Although a marked reduction in RBF is expected during the third trimester − 308 mL/min (range − 631 to − 117 mL/min), the value remains to be 30% (range 14–61%) higher than the measurement obtained from non-pregnant women [44–47]. Women investigated before pregnancy and after delivery showed no difference between measurements [45], indicating a return to normal values after childbirth [47].

Similar changes in RBF, but at smaller effect 8% (range 7–10%), were noted during the menstrual cycle [48]. In the luteal phase of the menstrual cycle, RBF measured using PAH clearance was found to increase on average by 77 mL/min (range 58–95 mL/min) [49]. This small variation in RBF was not detected in some studies using the same clearance method [50, 51].

Taken together, changes in RBF during normal pregnancy are substantial and if not considered can be confounded with hyperaemia. The impact of the menstrual cycle on RBF appears to be relatively small but controlling for this effect can inform a rigorous study design. In any case, querying information regarding menstrual health and history for female participants should be considered.

### Modifiable factors

#### Circadian cycle

A typical circadian cycle of renal haemodynamics exhibits a sinusoidal course [52], with peak and trough observed at late daytime wakefulness and night-time sleep, respectively [53]. Using the clearance technique, the average amplitude of change in RPF with day-night cycle was found to be 35% (range 25–52%) [52, 54–56]. A similar pattern was observed in global renal perfusion values, measured with ASL, which is more pronounced in the cortex than medulla [56]. In one study, no change in RPF was found when subjects were maintained at bed rest throughout the experiment [57], suggesting that circadian fluctuation of the renal circulation can be driven by behavioural stimulant such as changes in activity, food and fluid consumption during the day [58]. Nevertheless, the effect of circadian rhythms has been found to be persistent when identical meals were taken at regular intervals throughout the 24 h [3]. In line with these data, acquiring RBF measurement during a fixed time of the day should be considered to minimize the influences of daily cycle.

#### Food intake

Food intake is accompanied by profound cardiovascular changes in which hormonal and nervous systems are engaged to promote the digestion, absorption, and storage of nutrients in the body. Postprandial change in renal circulation is evident and depends on the size and macronutrient content of the meal.

The intake of high-energy fatty meal (142 g fat) was associated with a modest but sustained increase in RBF measured with PC-MRI of 23% after 20 min [59]. No change in RBF was observed after ingestion of mixed meal high in carbohydrate (> 90 g) in 1 h [60] or 4 h duration [61] using ultrasound.

After the ingestion of high protein meal, RBF measured with PAH clearance was found to increase by an average of 260 mL/min (range 150–390 mL/min) with peak values achieved 60–120 min postprandially [62–67]. The test meal used in these trials contained 80–90 g (1.2 g/kg) of different types of animal protein (including lean meat or dairy products) [64, 67]. Reducing the amount of protein (0.55 g/kg) appears to be associated with smaller renal response [68]. Two studies looked at RBF changes using PAH clearance in response to vegetarian protein, but the results are not aligned. One study showed no acute changes in RPF after consuming 80 g soy protein meal compared to meat meal in healthy subjects [69]. Another showed 14% increase in RPF values 90 min after intake of 77 g soy protein [70]. Given the small number of participants in both studies (*n* ≤ 10), it is difficult to conclude whether or not plant-based protein will elicit short-term changes in renal haemodynamics. The inconsistency between findings could be attributed to differences in the follow-up duration between studies, or heterogeneity in the soy protein supplement and the amino acid composition.

Studies investigating the chronic response of protein diet showed similar results to single-meal protein experiments. High protein diet (2.0 g/kg/day) was found to increase RPF compared to low protein diet (0.5 g/kg/day) when consumed for period of 6 days [71] or 3 weeks [72] in the adult kidney, but not in the elderly [73]. Switching from animal to vegetable-based protein diet for a period of 3 weeks was associated with lower resting RPF in healthy subjects [69, 74].

A trend towards higher RBF in individuals following high sodium diet (> 200 mmol/day or 11.7 g/day) compared to low sodium diet (50–70 mmol/day or 3–4 g/day) for period of 5–7 days was described in some [75–77], but not all studies [78, 79]. While PAH clearance was applied in all studies, the discrepancy might be explained by the amount of water intake which is known to enhance renal sodium excretion and hence modify the RAS-activity expected from a low sodium diet [80].

Since meal consumption is often associated with RBF changes, fasting before the study is commonly used. Low baseline RBF value was observed in some studies [63, 81, 82] in which the authors have attributed to overnight fasting, but no empirical study has yet sufficiently addressed this point. While awaiting further insight from future studies on the matter, a preliminary conclusion can be reached from the literature synthesis as to stress the importance of controlling food intake prior to RBF measurement.

#### Fluid intake/hydration

Water loading (20 mL/kg) induced a 10% increase in total RBF measured using PC-MRI [83], but no changes were observed in perfusion values of the cortex and medulla estimated using ASL [84]. In both studies, the renal response was evaluated relative to fasting conditions (i.e. fluid restriction). Comparing different hydration levels (high: 24 mL/kg vs. low: 3 mL/kg) using PAH clearance showed contradictory results. One study reported no change in RPF with different degree of water loading [85], while another found lower RPF (− 13%) in the higher hydration regimen [81]. Sampling error associated with the use of different methods for urine collection (spontaneous voiding [85] versus bladder catheterization [81]) imply that these findings should be interpreted with caution. A study conducted using ^131^I-hippuran scintigraphy showed that hydration did not affect the measurement of RBF, despite large inter-individual differences in RBF response [86]. The authors concluded that hydration can be advantageous as it aids rapid transport of radioactivity, and therefore peak activity can be achieved at an earlier time.

While the magnitude and direction of change in response to hydration status remains elusive, it can be inferred that acute physiological changes in RBF may appear in cases of sub-optimal or excessive hydration. There is no evidence against the regular intake of water which is in line with the recent consensus recommendation for PC-MRI [9].

#### Smoking, caffeine, and alcoholic consumption

Cigarette smoking has a profound systemic vasoconstriction effect associated with increased sympathetic stimulation and these responses are specific to nicotine component of the cigarettes. Chewing nicotine gum (4 mg) was associated with acute reduction (− 15%) in RPF in non-smokers [87, 88]. Habitual smokers, however, showed impaired renal response following active smoking (2–3 mg nicotine) [89], suggesting the development of nicotine tolerance in these individuals compared to non-smokers [87]. Comparing chronic smokers and non-smokers showed a lower RPF (− 23%) in the smoking group [90], and steeper decline in RPF with age in smoking male subjects [91].

Few studies have looked at the effect of caffeine and alcohol consumption, despite their well-known diuretic effect. One study using PAH clearance found no change in RPF after consuming beer (666 mL) or water containing 2.7% alcohol [92]. Similarly, caffeine intake had no acute effect on PAH clearance when consumed in doses less than 400 mg [93, 94]. There were no reports concerning the impact of regular (chronic) consumption of alcohol, caffeine, or other recreational drugs on RBF in humans.

At present, the limited data on the potential effect on RBF, preclude restricting the acute use of nicotine, alcohol, or caffeine substances. Future studies are required to confirm these findings. Nevertheless, querying the habitual use of these substances should be considered to support the interpretation of RBF results [90].

#### Exercise

The renovascular adjustments to exercise training occur in response to the increased sympathetic neural outflow, resulting in blood flow being directed away from the kidneys [95]. Using PAH clearance, a substantial decline − 40% (range − 26 to − 58%) in RBF − 450 mL/min (− 279 to − 759 mL/min) was reported immediately following dynamic exercise [96–100]. Similar response was observed in renal perfusion of the cortex [101–103] and medulla [103], measured using PET [101, 102] and MRI [103], following static handgrip exercise. A return to near pre-exercise RBF value can appear 30–60 min after completion of the dynamic exercise bouts [2, 104], with full-recovery reported at 2-h post-activity [105]. In older adults, changes in RBF during exercise is lower [106, 107] and recover at slower rate [106, 108]. Studies investigating the effect of variable level of exercise training showed minimal RBF response with low exercise intensity [99, 100, 109, 110]. The degree of RBF reduction can also be related to other factors such as exercise duration [100], ambient temperature [97, 99, 107], and hydration level [111].

Difference in resting RBF measurements between sedentary and well-trained young individuals was noted in a cross-sectional study [107]. Nevertheless, no effect on RBF was found when subjects underwent a sustained exercise training program for 4 weeks [107] or 6 months [112].

The transient change in RBF post-exercise might be confounded with reduced renal function. Hence, heavy physical activity should be restricted on the day of RBF measurement.

#### High altitudes

Exposure to high altitude is associated with an elevation in haemoglobin concentration and a redistribution of RBF which serve to restore normal blood oxygenation as a consequence of acute hypoxia. RBF measured with PAH clearance [113–115] or Doppler ultrasound [116] showed a reduction of − 17% (range − 10 to − 23%) in response to short-term (1–7 days) exposure to high altitude (range 3500–6500 m). The reduction in RBF is maintained during prolonged stay (> 60 days) [115] but was not detected with shorter exposure time (6 h) using Doppler US [117]. Natives living at high altitude have also been found to have − 28% lower RBF values when compared with sea level residents [118, 119]. While the impact of altitude adaptation might be less relevant in single-centre studies, it should be considered when comparing data that involve individuals residing at high-altitude environment.

#### Mental activity

The renal response to acute mental stress is characterized by a rapid and transient vasoconstriction stimulated by sympathoadrenal excitation. Cortical [120] and total RBF [121–123] was found to decrease − 19% (range − 6 to − 33%) in response to solving stressful mental arithmetic [123] or the Stroop colour-word tests [120–122]. Alterations in RBF were observed at 2 min of mental stress [120] which would gradually return to baseline at 1 h [122]. The reduction in RBF can be negatively associated with the increment in systolic blood pressure during mental stress [123]. In the elderly, mental stress results on more pronounced and prolonged reduction in RBF [124]. Similar changes were noted in renal circulation following emotional stress induced by the discussion of sensitive personal topics [125]. In addition, individuals annoyed by environmental noise were found to have 10% lower RBF compared to non-noise-annoyed individuals [126].

These data support that anxiety, tenseness, and annoyance are factors that should be considered (and possibly controlled) when measuring RBF. Ensuring the participant feels comfortable before and during the scan can be achieved by thorough explanation of the procedure. Since the MRI scanner environment can be an obvious source of apprehension to some participants (i.e. claustrophobia), it is advised to record for such effects which would aid the interpretation of results. In addition, capturing information on individual’s feelings (i.e. anxiety and depression) should provide additional comparative data between subjects.

#### Thermal stress

Exposure to thermal stress causes a redistribution of RBF where the kidney acts to maintain internal body temperature by activating the sympathetic outflow. Reduced RBF measured with PAH clearance − 31% (range − 26 to − 38%) was reported in response to increased body temperature + 0.5 to + 2 °C following exposure to hot environment (50 °C dry bulb) [97], or passive heating induced with water-perfused suits [127–129]. RBF reduction was only minimal when subjects were exposed at a milder air temperature (36 °C) [99], or internal body temperature rose by 0.4 °C [99, 130]. Sustained heat exposure (i.e. heat acclimation) at 30 °C for period of 4 days had no measurable effect on baseline RBF [131]. The cooling effect provoke similar renal response. One study using ASL reported reduced renal perfusion in the cortex but not in the medulla when participant’s feet were covered with ice packs at 1 °C for 2 min [132]. This effect has similarly been observed on ultrasound measured renal blood velocity and resistance index [133–135]. The pattern that emerges from these studies is that changes in body temperature is associated with large RBF fluctuation. Measurement of body temperature should therefore be obtained, and the study can be delayed in case of fever.

#### Medication use

Extensive research has been performed to investigate the alterations of renal circulation in response to a wide range of pharmacological treatments. In this section, an emphasis will be given to the use of commonly prescribed medications that have been studied in normal human subjects.

*Nonsteroidal anti-inflammatory drugs (NSAID)* can inhibit prostaglandin synthesis, thereby causing important alterations in renal function. Studies on normal subjects have reported variable RBF response of selective and non-selective NSAIDs.

The oral administration of indomethacin (50 mg) was found to acutely reduce RPF − 16% (range − 10 to − 23%) measured using PAH clearance in young normotensive subjects who were maintained on sodium balance [136–138]. However, the short-term intake of indomethacin for period of 3–7 days resulted on preserved RPF both in young [139–141] and elderly subjects [142].

Under conditions of severe salt depletion, single oral dose of celecoxib (400 mg) resulted on a transient drop in RPF measured with PAH clearance within 1 h [143], but no changes were observed in the same study when a lower dose (200 mg) was used. This reduction in RPF with the higher dose of celecoxib (400 mg) was not observed in a separate study performed on subjects with minimal salt depletion [144].

A daily dose of diclofenac (50 mg) used for period of 2–3 days had no alteration in RPF measured using standard PAH clearance both in young [145] and older patients without impaired renal function [146]. Similarly, using ASL no changes in renal perfusion were detected after single oral dose (50 mg) or short-term tropical application (3 days) of diclofenac [147]. However, a significant reduction in renal perfusion was reported, in the same study, in sub-group of subjects with high plasma diclofenac level after oral intake only.

Previous studies that have investigated the effect of ibuprofen (400–800 mg) on renal circulations reported no significant changes in RPF after acute [137, 148], short-term (3 days) [141] or sustained (14 days) [149] administration in salt-replete subjects. Similarly, therapeutic doses of etodolac (300 mg) [149], paracetamol (500 mg) [140], Aspirin (975 mg) [137, 150], diflunisal (500 mg) [137], naproxen (500 mg) [143] or ketoprofen (50 mg) [139] for short-term did not affect RPF in healthy young subjects.

These data indicate that various NSAIDs have different effects on RBF in normal individuals. It can also be inferred that the changes in RBF are more pronounced during acute oral use, rather than the repeated use of NSAIDs, possibly due to counterregulatory mechanism operating in the human kidney. In addition, the specific effect of NSAIDs can be confounded by the state of sodium intake, with more pronounced effect expected during sodium-restriction [151]. Taken together, participants can be advised to avoid NSAIDs before RBF measurement, and recent use of NSAIDs should be documented.

The use of *oral contraceptive pills (OCP)* is known to be associated with increased RAS activity, but their impact on renal hemodynamic has been controversial. An earlier study reported reduction in renal perfusion by an average of − 25%, estimated from the disappearance curve of radioactive xenon, in normotensive women using a variety of combined oestrogen-progestogen drugs for long-term (> 6 months) [152]. Similar, though minor, differences were observed when comparing baseline RBF between OCP users (30 ± 5 µg ethynyl-oestradiol) and non-users in some [153, 154], but not all studies [155, 156] using PAH clearance. The progestational and androgenic activity in the OCP has been found to be associated with enhanced angiotensin-dependent control of the renal circulation [157]. The discontinuation of OCP for period of 6 months had no measurable effect on baseline RBF [155]. These data suggest that the regular use of OCP should be documented when measuring RBF in women.

*Renin-angiotensin system (RAS) inhibitors*, reduce the formation of angiotensin-II, which results on reduced systemic and renal vascular resistance and favourable renal vasodilator effect. Different classes of RAS inhibitors have been studied previously namely, angiotensin converting enzyme (ACE) inhibitors, angiotensin receptor blockers (ARB) and direct renin inhibitors. In spite of differences in their mechanism of actions, the acute administration of these agents has been found to increase RBF to variable degree.

In healthy subjects, the acute administration of Captopril (25 mg) caused marked rise in RPF (range 14–24%) [148, 158] with peak values reported at 3–4 h [158]. Two ascending doses of Ramipril (2.5 mg and 10 mg) administrated on separate days resulted on similar increase in RBF, with an estimated change of 18% reported with the highest dose during 3.75–4.75 h period [159]. Treatment with Enalapril (20 mg) caused modest increase in RPF (range 10–13%) at 2–4 h [160, 161], but maximal vasodilation was noted 6 h after drug intake [160].

Studies using drugs of the ARB class showed similar renal hemodynamic effect to those using ACE inhibitor [162]. In healthy, salt depleted subjects, single oral dose of Eprosartan (200 mg) increased baseline RPF by 20% at 3.75 h post-administration [163]. In the same study, the lowest Eprosartan dose sufficient to cause significant renal vasodilation was determined to be < 10 mg, whereas the near-maximal vasodilator response was achieved with a dose of 100 mg. In similar laboratory sittings, escalating doses of Candesartan caused progressive increase in RPF with peak response (24%) achieved with a dose of 16 mg during 4 h after administration [164]. Studies using Valsartan (80 mg) [160] or Losartan (50 mg) [165] on healthy subjects showed relatively small increase in RBF (range 8–11%) during 3–4 h after acute administration.

One study looked at the effect of direct renin inhibitor, Aliskiren, and found marked dose-related renal vasodilation [166]. Under conditions of salt-depletion, a change from baseline RPF values of 16%, 22% and 30% were reported in repones to acute aliskiren doses of 75 mg, 150 mg, and 300 mg, respectively. In addition, baseline RPF remained significantly increased after each aliskiren dose when repeated measurements were performed 48 h post-drug intake [166].

*Calcium channel blockers* represent another class of drugs that have been associated with a preferential vasodilation effect on afferent arterioles. Normal subjects receiving two separate doses of felodipine (5 mg and 20 mg) showed major changes in RBF acutely, with maximum estimated change of 40% reported with the higher dose [159]. On the other hand, the acute use of Nifedipine (10 mg) caused minimal vasodilation in baseline RPF value [158]. This considerable variability in the renal hemodynamic effects among calcium antagonists, might be related to differences in the intrinsic actions of the agents studied.

*Beta blockers* suppress cardiac output and inhibit mediated vasodilation, both effects could result in reduced RBF. However, previous studies reported conflicting results which make the effect of beta blockers difficult to interpret. One study using PAH clearance showed the anticipated reduction in RBF (-11%) in the first hour following oral intake of metoprolol (100 mg) or pindolol (10 mg) in hypertensive patients with normal kidney function [167]. An opposite trend was reported in response to the same dose of metoprolol in a separate study performed on healthy volunteers. No changes in baseline RBF were reported following 10 days treatment with metoprolol (200 mg) compared to placebo [168].

In general, anti-hypertensive medications represent potent modulators of RBF. Patients with prescribed anti-hypertensive treatments (RAS inhibitors, calcium channel blockers, beta blockers) can be advised to withdraw acute use of drugs before RBF measurement where possible.

*Diuretics* can affect renal haemodynamics through their actions as an inhibitor of sodium-reabsorption in the ascending loop of Henle. One study, using PAH clearance, compared the effect of oral intake of four different loop diuretics on healthy volunteers and reported no alteration in RPF in response to piretanide (6 mg) or bumetanide (1 mg), whereas ethacrynic acid (100 mg) and furosemide (40 mg) induced reduction in RPF by − 23% and − 7%, respectively [169]. Variable effects have been found in response to the acute parenteral administration of furosemide in healthy volunteers. Two studies, using PAH clearance, reported transient increase in RPF in the first 20 min after furosemide (20 mg) injection [141, 170]. Other studies applying PAH clearance [171] or PC-MRI [103] reported no alteration in RBF post-furosemide injection. Regional perfusion in the cortex and medulla, estimated using ASL, have been found to decrease post-furosemide injection [172].

While the inconsistency between findings makes the net effect of loop diuretics difficult to conclude, omitting diuretic drugs in the morning before RBF measurement should be considered where possible.

## Discussion

This review has evaluated the magnitude and direction of change of several influencing factors on RBF with the goal to formulate evidence-based recommendations. The results obtained from the literature support the need for a rigorous study design to enable more efficient quantification of RBF. Despite some large variability in the reported magnitude of change, the direction of change was mostly aligned between studies. It must be noted that measurement error is inevitable and constitute an additional source of variability, even if a gold-standard method was used.

Standardization is a pivotal initial phase in biomarker validation. Ongoing efforts for standardisation of renal MRI seek to establish consistent and reliable measurement protocols [8–10]. The work presented in this review paper supplement existing recommendations with additional evidence-based information and could promote an alignment of opinions in future revisions of the consensus. A wide range of influencing factors was found to cause changes in RBF. In the recently published technical recommendations for PC-MRI, the panel has advised against restricted diet and hydration (i.e. fasting), instead scans are recommended to be performed in the normal hydration state while avoiding salty- and protein rich meals [9]. Although there is a dispute on the acute effect of water loading, hydration is undeniably a standard procedure used in clearance studies to maintain urine flow at constant rate. At present, the provided evidence supports the relevance of normal hydration before RBF measurements. With respect to food intake, our recommendation is less clear-cut, and calls for future research in this matter.

Depending on the influencing factor, RBF can decrease by a mean of 130 mL/min or increase by 250 mL/min. Differences of this magnitude have an important clinical implication and can be confounded with disease characteristics. For instance, patients with benign hypertension show reduced RBF compared to controls subjects [173]. An effect of similar magnitude and direction can be observed post exercise or in response to heat exposure. By contrast, newly diagnosed diabetes is characterized by higher RBF than in normal subjects [174, 175]. An effect of similar pattern can be observed in response to protein meal in healthy subjects [67].

Considering the data presented in this review, we propose evidence-based recommendations to provide future researchers a means to interpret and possibly correct for the influencing factors. While the review primarily aimed at assessing RBF and perfusion, the proposed recommendation might also apply to other techniques such as renal BOLD MRI. The specific recommendations are summarized in Table 3, and include all factors, even those only minimally contributing to the variability. The physiological confounders were arranged into three separate categories in relation to the stage where they should be considered. Since it may not be feasible to control for all modifiable in clinical practice, it can be advised to acquire information using questionnaire. For instance, providing a standardized meal before conducting the exam can ensure reducing the influence of certain type of food on RBF, but may also cause financial and practical constrains. An alternative option would be to ask participants to keep a record of their last meal, which can be referred to in case of unusual measurement. While current evidence in the literature showed preserved RBF in response to acute use of caffeine, nicotine and alcoholic products, more studies would be helpful to support this conclusion. Our recommendation to withdraw acute medications was based on current preparation worked in a clinical trial [176]. However, whether this acute cessation of treatment has an impact on renal haemodynamics was not studied before. Similarly, little is known about the acute use of some novel medications, notably sodium–glucose cotransporter 2 (SGLT2) inhibitors and glucagon-like peptide 1 (GLP-1) receptor agonists. These medications have been found to contribute to improved renal outcomes [177], and more comprehensive investigation into their acute influence on RBF is needed to understand their potential implications. As a general rule, obtaining additional information regarding individual habits, and/or prescribed medications can be considered for a rigorous study design. These factors can then be used as covariates in the statistical analysis model to enable better interpretation of RBF measurement.

### Limitations

One limitation of this review arises from the inclusion of studies using various techniques to measure RBF in response to each influencing factor. The unit of RBF was inconsistently reported in the literature, where normalization to BSA was used in some but not all studies. For the analysis of this review, RBF was assessed in absolute units (i.e. mL/min) as correcting to BSA is a potential confounder and therefore can obscure some of the variation between individuals of different body dimensions. It must therefore be emphasized that the magnitude of change in absolute units presented in this review should not be used to correct for such effects but rather as an estimation of the effect size. A detailed discussion of the underlying physiological mechanism affecting an individual confounding factor would enable better understanding of the reported results, but this was not the main objective of the current review.

## Conclusions

The variability in RBF in response to physiological factors is an important consideration in the development of an efficient study design to assess the renal hemodynamic. In this review, the effect of several influencing factors was assessed in order to provide an evidence-based recommendation. Future studies aiming to measure RBF are encouraged to follow a rigorous study design, which takes into account the factors that can influence RBF results. In addition, some gaps in the scientific literature were highlighted that represent exciting opportunities for future exploration to expand our understanding of kidney physiology and more importantly, promote well-controlled quantitative assessment.

## Data Availability

Data sharing is not applicable to this review article as no new data were created or analyzed in this study.
